# Elevated Blood Lead Levels Are Associated with Reduced Risk of Malaria in Beninese Infants

**DOI:** 10.1371/journal.pone.0149049

**Published:** 2016-02-11

**Authors:** Violeta Moya-Alvarez, Michael Osei Mireku, Pierre Ayotte, Michel Cot, Florence Bodeau-Livinec

**Affiliations:** 1 Institut de Recherche pour le Développement (IRD), Mère et Enfant Face aux Infections Tropicales (UMR 216-MERIT), Paris, France; 2 Université Pierre et Marie Curie (UPMC- Paris VI), Ecole doctorale Pierre Louis de Santé Publique, Paris, France; 3 Réseau doctoral de l'Ecole des Hautes Etudes en Santé Publique, Rennes, France; 4 Ecole des Hautes Etudes en Santé Publique, Département Épidémiologie et Biostatistiques, Rennes, France; 5 Département de médecine sociale et préventive, Université Laval, Québec, Canada; 6 Axe Santé des populations et pratiques optimales en santé, Centre de recherche du CHU de Québec, Québec, Canada; 7 Direction de la santé environnementale et de la toxicologie, INSPQ, Québec, Canada; 8 PRES Sorbonne Paris Cité, Université Paris Descartes, Faculté des Sciences Pharmaceutiques et Biologiques, Paris, France; 9 Inserm UMR 1153 Obstetrical, Perinatal and Pediatric Epidemiology Research Team (Epopé), Center for Epidemiology and Statistics Sorbonne Paris Cité, DHU Risks in pregnancy, Paris Descartes University, Paris, France; Centro de Pesquisa Rene Rachou/Fundação Oswaldo Cruz (Fiocruz-Minas), BRAZIL

## Abstract

**Introduction:**

Elevated blood lead levels (BLL) and malaria carry an important burden of disease in West Africa. Both diseases might cause anemia and they might entail long-term consequences for the development and the health status of the child. Albeit the significant impact of malaria on lead levels described in Nigeria, no evaluation of the effect of elevated BLL on malaria risk has been investigated so far.

**Materials and Methods:**

Between 2010 and 2012, blood lead levels of 203 Beninese infants from Allada, a semi-rural area 50km North from Cotonou, were assessed at 12 months of age. To assess lead levels, blood samples were analyzed by mass spectrometry. In parallel, clinical, microbiological and hematological data were collected. More precisely, hemoglobin, serum ferritin, CRP, vitamin B12, folate levels, and *Plasmodium falciparum* parasitemia were assessed and stool samples were also analyzed.

**Results:**

At 12 months, the mean BLL of infants was 7.41 μg/dL (CI: 65.2; 83), and 128 infants (63%) had elevated blood lead levels, defined by the CDC as BLL>5 μg/dL. Lead poisoning, defined as BLL>10 μg/dL, was found in 39 infants (19%). Twenty-five infants (12.5%) had a positive blood smear at 12 months and 144 infants were anemic (71%, hemoglobin<110 g/L). Elevated blood lead levels were significantly associated with reduced risk of a positive blood smear (AOR = 0.38, P-value = 0.048) and *P*. *falciparum* parasite density (beta-estimate = -1.42, P-value = 0.03) in logistic and negative binomial regression multivariate models, respectively, adjusted on clinical and environmental indicators.

**Conclusion:**

Our study shows for the first time that BLL are negatively associated with malarial risk considering other risk factors. Malaria is one of the main causes of morbidity and mortality in infants under 5 years worldwide, and lead poisoning is the 6th most important contributor to the global burden of diseases measured in disability adjusted life years (DALYs) according to the Institute of Health Metrics. In conclusion, due to the high prevalence of elevated BLL, health interventions should look forward to minimize the exposure to lead to better protect the population in West Africa.

## Introduction

Elevated lead level has severe harmful effects on infant health. Symptoms related to toxicity occur from mid to high levels of exposure and they depend on the amount of lead in the blood and tissues. High lead levels are associated with impaired neurocognitive development, anemia (due to either disruption of heme synthesis or hemolysis[1]), and renal and gastro-intestinal effects[2]. Although high blood lead levels (BLL) (BLL >100 μg/dL) can entail acute neurologic symptoms, such as ataxia, hyperirritability, convulsions, coma, and death, BLL as low as 10 μg/dL have been also correlated with poor neurocognitive outcomes and behavioral disorders[3,4]. This is of special concern in young children as neuro-cognitive impairment has been found to be associated with the degree of exposure to lead between the ages of 12 and 36 months[5]. Indeed, the Center for Disease Control (CDC) reduced the reference level of blood lead from 10 μg/dl to 5 μg/dl [6] in 2012.

Albeit the severe impact of elevated lead level on infant health, epidemiological studies of lead level in Sub-Saharan Africa are limited. Data from the few existing studies, published in a systematic review on BLL among Sub-Saharan children, suggest an alarming burden of elevated BLL. This review reported a BLL weighted mean of 13.1 μg/dl which increased up to 16.2 μg/dl considering solely studies with robust quality BLL analyses[7]. In addition, the prevalence of BLL >10 μg/dL exceeded 44% in all cases reviewed, with a maximum of 70.9% in Nigeria. Only one study in Kenya reported a relatively low prevalence (7%). Recent mass level intoxications reported in Senegal and Nigeria[8] further raise the public health concern about lead exposure in West Africa.

In addition, malaria and lead poisoning overlap geographically. Indeed, infectious diseases, mainly malaria, dominate the disease burden in West Africa[9]. In Benin, malaria is the main cause of mortality among children less than 5 years and there were over 1.5 million cases in 2012[10]. As already explained, both malaria and lead poisoning can have severe hematologic and neurologic symptoms on children and their development. Malaria and lead poisoning may not only overlap, but they have major impact on the health of children, especially those under 5 years. Consequently, their possible association may have an effect on one of the most vulnerable age groups in the population, and it could have severe long-term implications for the development of the children. Furthermore, Nriagu found a significant effect in univariate analysis of malaria on the children lead levels in different areas of Nigeria[11]. However, this article refers only to malaria episodes recalled by the children or their family during the previous 6 months and no biological evaluation of malaria is conducted in his study. Furthermore, malaria is not included in the multivariate analysis of factors associated with BLL. Indeed, no accurate evidence exists at present on the possible joint effect of lead and *P*.*falciparum*. To our knowledge, no published study exists on lead levels in Benin, and in particular, on the effects of lead levels on malaria risk in infants. Therefore we aim at analyzing the effect of lead levels on malaria risk with regard to both the possibility of having a positive smear and their effect on *P*.*falciparum* parasite density taking into account hematological and parasitological factors.

## Materials and Methods

Our cross-sectional study used data obtained from two-hundred and three infants at 12 months of age. These infants were followed from birth until 12 months of age in two embedded studies: the APEC study (Anemia in Pregnancy: Etiology and Consequences) and the TOVI study. The TOVI study evaluated the children for cognitive and motor functions using the Mullen Scales of Early Learning as well as their lead levels at 12 months[12]. The APEC study involved a prospective cohort of 400 infants followed from birth to 12 months of age to assess their health status. The follow-up of the APEC study was mainly focused on the malarial and hematologic status of the infants. Indeed, malaria detection was performed systematically at birth, at 6, 9, and 12 months of age. In addition, passive case detection was performed continuously during follow-up. Malaria history of their mothers during pregnancy is well described in an article already published[13]. The 203 infants of our sample correspond to the infants for whom data at 12 months include a complete follow-up of lead and malaria indicators.

For further details, APEC study is an ancillary survey nested within the MiPPAD study in Benin (Malaria in Pregnancy Preventive Alternative Drugs “http://clinicaltrials.gov/ct2/show/NCT00811421”). This study was conducted in three clinics in the district of Allada (Allada, Attogon, Sékou), between January 2010 and May 2012. Allada is a semi-rural area of 91,778 inhabitants located 50 km North of Cotonou (Benin). Malaria has a perennial transmission pattern with two transmission peaks corresponding to the rainy seasons in April-July and October-November. *Plasmodium falciparum* is the species responsible for the majority of infections.

At 12 months, clinical data of the infants were collected, including anthropometric measures and clinical examination. Weight was measured using an electronic baby scale (SECA type 354) with a precision of 10 g and length was measured to the nearest 1 mm with a locally manufactured wooden measuring scale according to the criteria recommended by WHO. Eight milliliters (mL) of venous blood were obtained from each participant. Hemoglobin, serum ferritin, CRP, vitamin B12, lead and folate levels were thereby assessed. A container was also given to the women to collect stools to examine the presence of intestinal helminths in the infants. Microbiological exams were realized as follows: Lambaréné technique was used to assess malaria infection on thick blood smears [14]. It consists of spreading a calibrated 10 μL amount of blood on a slide’s rectangular area of 1.8 cm2 (1.8 x 1 cm). The slide was stained with Giemsa and read at a magnification of 1,000 × with an oil immersion lens. To assess parasite density (in parasites/μL), a multiplication factor was applied to the average parasitaemia/field. Helminthic infestations were assessed using the Kato-Katz concentration method (VestergaardFrandsen kit^®^). The hemoglobin level was measured with a Hemo-Control photometer (EKF Diagnostics, Magdeburg, Germany) device. Serum ferritin, folic acid, and vitamin B12 concentrations were measured using a microparticle enzyme and fluorescence polarization immunoassay (AxSym Immuno-Assay Analyzer, Ab- bott Laboratories). CRP concentration was determined by rapid slide test (CRP Latex; Cypress Diagnostics Inc.) to correct the effect of inflammatory syndromes on ferritin concentrations. More precisely, we corrected serum ferritin in the context of inflammation following the procedure inspired by the meta-analysis by Thurnham[15] before conducting the analyses, so we multiplied serum ferritin by 0.76 in the presence of *Plasmodia* without inflammation, and we multiplied serum ferritin by 0.53 in case of concurrent *Plasmodia* infection and inflammation. Iron deficiency was then defined as corrected serum ferritin concentration <12 μg/l in infants. Iron deficiency anemia (IDA) was defined as hemoglobin<110 g/l with iron deficiency.

With regard to BLL, 4 mL (out of the 8 ml obtained at 12 months) were collected into a tube containing dipotassium EDTA and 4 mL into an iron-free dry tube. Blood samples were analyzed for lead using method M-572 of INSPQ’s Toxicology Laboratory, which is accredited ISO 17025 and participates in the QA/QC program of the Canadian Northern Contaminants Program and the Arctic Monitoring Assessment Program. Briefly, samples were diluted 20-fold in ammonia 0.5% v/v and 0.1% v/v Triton-X 100 and analyzed by inductively coupled plasma mass spectrometry (ICP-MS; Perkin Elmer Sciex Elan DRC II ICP-MS instrument). The limit of detection was 0.2 μg/L and inter-day precision was 7.5% at 14 μg/L. The samples were transmitted by DHL frozen in icepacks directly from Cotonou to the University of Laval.

Preventive measures against malaria in the APEC study include insecticide treated bednets (ITNs) and intermittent preventive treatment in pregnancy (IPTp). Infants were treated with artemether-lumefantrine in the case of malaria according to Beninese guidelines.

Because of the anopheline breeding cycle, the mean rainfall volume of the 7 days prior to the two weeks before the consultation was calculated. It was independently assessed for each health centre of the district of Allada.

Socio-economic status was assessed using a socio-economic index created in a two-step process. First all socio-economic items (home possession of latrines, electricity, a refrigerator, a television, a vehicle with at least two wheels, being married, and working outside the home) were plotted into a multiple correspondence analysis[16,17]. Then, two predictors were created to synthesize the information, and as the first captured the large majority of the information, it was withheld as the socio-economic index. We used this approach because it allows us to create a synthetic objective index of socio-economic items without any a priori on the weight of each of the elements of the index.

### Statistical analysis

Data were double entered and analyzed with *ACCESS2003* and *STATA12*.*0* softwares for Windows (Stata Corp, College Station, TX, USA). Univariate analysis was performed to assess the association of all variables with either the infant positive smear or peripheral *P*.*falciparum* density at the moment of lead assessment (at 12 months of age). Thereafter, all variables with *P* values<0.2 were included in a multivariate model regression. Logistic regression was used to evaluate the determinants associated with a positive blood smear. Negative binomial regression was used for the multivariate analysis of *P*.*falciparum* parasite density. Lead level was analyzed as a variable with different categories as there is no linear relationship between parasite density and lead levels. Socio-economic status was forced into the model because of its known association with lead levels according to the literature[11]. The statistical significance in the final multivariate models was set to *P*<0.05.

### Ethical considerations

This study and the consent procedure regarding the women and their offspring were approved by the Ethics Committee of the Faculty of Medicine of Cotonou, Benin. It was explained in local language to the participant and her voluntary written consent was obtained and recorded in the clinic files before enrolment. In case the woman could not read, an impartial witness was included in the process. In the case of the inclusion of minor women, both their consent and the consent from the parents or legal guardians were obtained. Women were free to interrupt their participation at any time of the study. In addition, the study was also approved by the ethics committee of the New York University, in the context of the NIH funding of the TOVI study.

## Results

**Fig 1:**
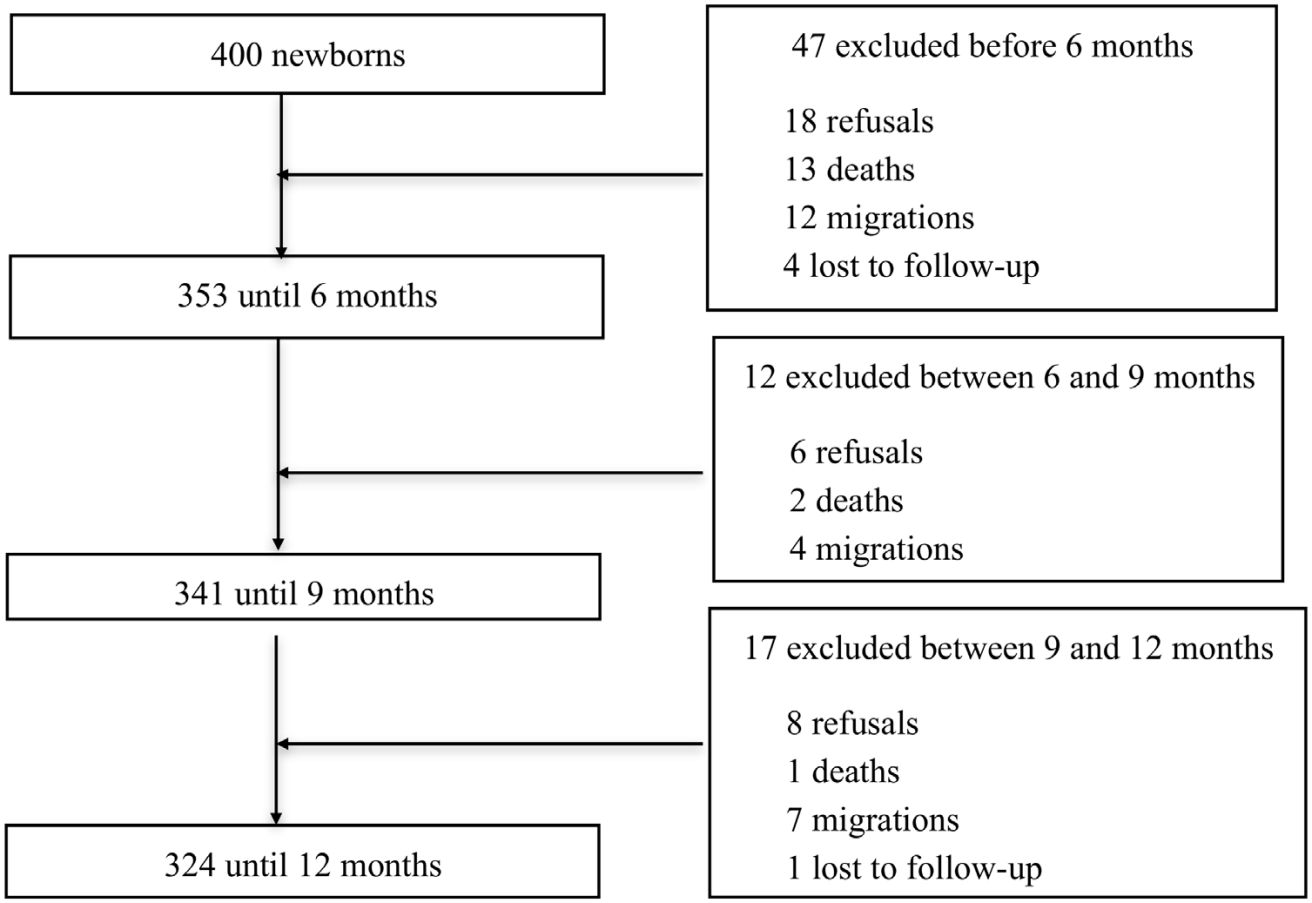
Infant follow-up.

The BLL of 203 infants included in the APEC-cohort were obtained at the 12-month visit between April 2011 and May 2012. During the 12-month follow-up 84 infants (42%) had at least one malarial episode. More precisely, 60.25% of infants had no positive blood smear during the entire follow-up period, 22% of infants had 1, 12.50% had 2, 4.5% had 3, and 0.75% had 4 positive blood smears during follow-up. The main demographic, malarial and hematological indicators as well as lead levels are presented in Table 1. There was a majority of girls, and, on average, the infant’s weight was 8.44 kg and their height was 72.56 cm. This corresponds to a small weight for height index compared to WHO standards. There were 76 infants excluded during the 12-month follow-up, including 5 lost of follow-up (Fig 1). There were no differences with regard to malaria risk and the characteristics at enrolment between the infants excluded and the infants that completed the follow-up. In addition, multiple imputation technique was used and results did not differ significantly.

**Table 1:** Demographic and clinical characteristics of the infants.

Parameters	Mean or number of people affected
Gender	Male: 96 (48.48%), Female: 102 (51.52%)
Weight (g)	8437.25 (CI:8285.02; 8589.48)
Height (cm)	72.56 (CI: 72.04; 73.06)
Malaria infection (%)	25 (12.5%)
*P*.*falciparum* density (parasites/μL only for infants with positive smears)	13460 (CI:2775; 24145)
Blood lead levels (μg/L)	74.1 (CI: 65.2;83)
Elevated blood lead levels (BLL>5 μg/dL)	128 (63.05%)
Lead poisoning levels (BLL>10 μg/dL)	39 (19.21%)
Haemoglobin (g/L)	101.69 (CI: 99.51; 103.86)
Anaemia (Hb <110 g/L)	144 (70.94%)
Ferritin (mg/L)	571 (CI: 429.67; 712.34)
Iron deficiency (corrected SF <15 μg/L)	85 (42.93%)

**Fig 2:**
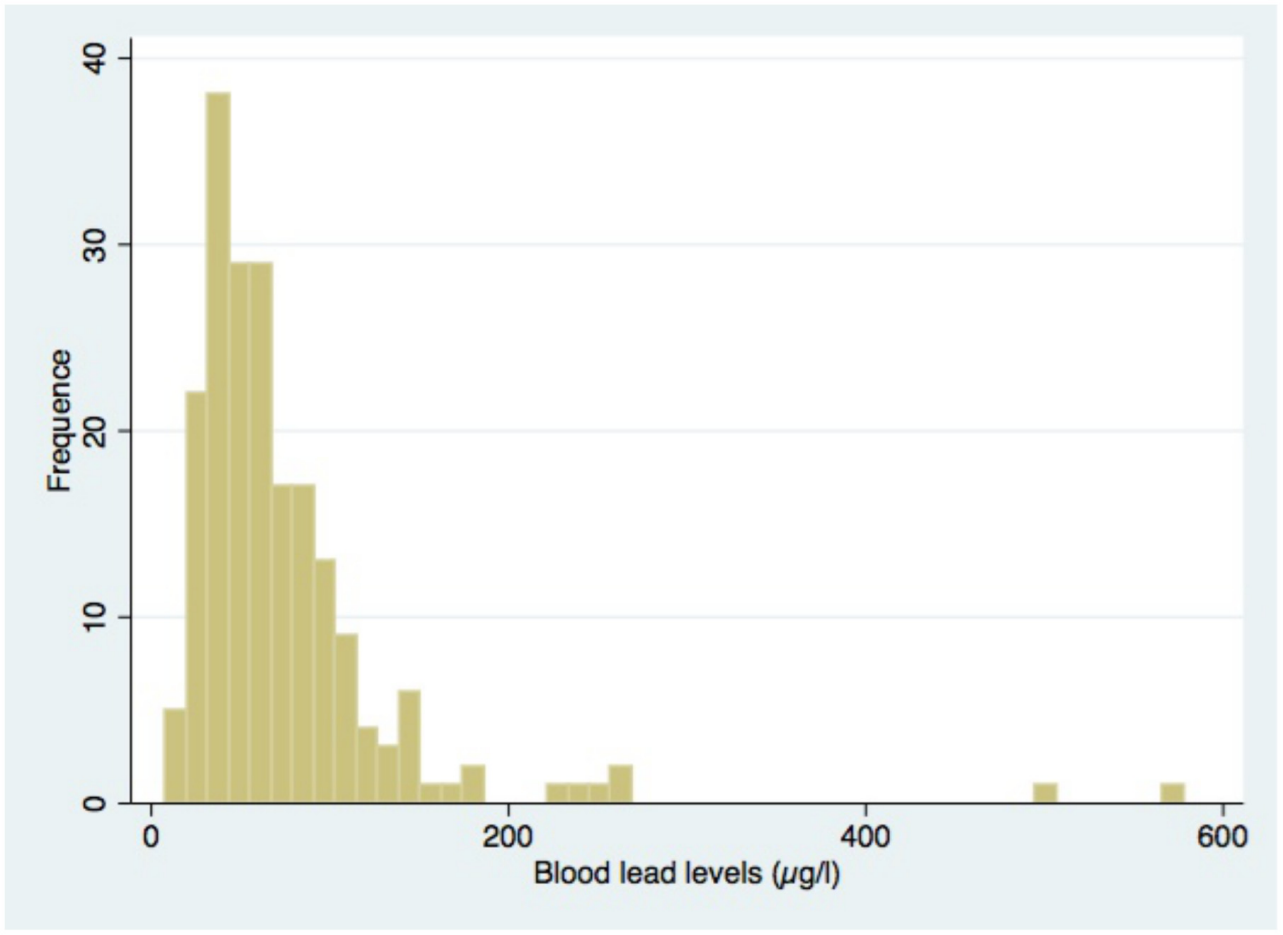
Distribution of the infant blood lead levels at 12 months.

At the moment of lead assessment, 25 out of 200 (12.5%) of the infants had a positive blood smear, with a mean parasite density of 13460 (CI: 2775; 24145). Lead levels were high overall. The mean BLL of infants was 7.41 μg/dL (CI: 65.2; 83), and 128 infants (63%) had elevated blood lead levels, defined by the CDC as BLL>5 μg/dL. Lead poisoning, defined as BLL>10 μg/dL, was found in 39 infants (19.21%). The distribution of blood lead levels is presented below (Fig 2). More concretely, it corresponds roughly to a approximately to a shape of a log-normal distribution. The lead distribution function can be divided in 4 quartiles that gather the different BLL values of the infants. The minimum of the function corresponds to an infant with BLL at 0.83 μg/dL and the infant with the highest BLL has 58.7 μg/dL. More precisely, the 1^st^ quartile includes infants with BLL between 0.83–3.9 μg/dL, the 2^nd^ quartile includes infants with BLL between 3.9–5.8 μg/dL, the 3^rd^ quartile includes infants with BLL between 5.8–8.7 μg/dL, and the 4^th^ quartile includes infants with BLL between 8.7–57.8 μg/dL.

With regard to the hematological indicators, 144 infants were anemic (70.94%, hemoglobin<110 g/L), and 85 were iron deficient (42.93%, CRP-corrected serum ferritin (SF) <15 μg/L). The mean and median hemoglobin and ferritin values were 101.69 g/L and 104 g/L (CI: 99.51; 103.86), and 571 mg/L and 201.5 mg/L (CI: 429.67; 712.34), respectively.

In both univariate and multivariate analyses, malaria risk and parasite density were treated as the dependent variables. Weight, height, temperature, hemoglobin, serum ferritin, CRP, vitamin B12, folate, blood lead levels, helminthic infestations, socio-economic status, and rain volume were included in the analyses as independent variables and considered in both analyses.

At 12 months, ferritin, vitamin B12 and CRP levels as well as low socio-economic status were associated in univariate analysis with increased malaria risk with regard to both the risk of having a positive smear and *P*. *falciparum* parasite density (Table 2). Rain volume was borderline associated with malaria risk (p-value = 0.06). In parallel, hemoglobin and lead levels were inversely correlated with malaria risk in univariate analysis. For both univariate and multivariate analyses all variables measured in the TOVI and the APEC study, described in the methods section, were considered. Only statistically significant variables or variables important for malaria risk according to literature (in this case the socio-economic status) were kept in the final models.

**Table 2:** Univariate analyses of variables associated with malaria risk at 12 months.

Variables	p-value
Blood lead levels	0.001
Hemoglobin	0.001
Ferritin	0.01
Low socio-economic status	0.01
CRP	0.045
Vitamin B12	0.05
Rain volume	0.06

There were no statistical significant differences in malarial, lead, or hematologic indicators depending on the health care centre. Tables 3 and 4 describe risk factors associated with the possibility of having a positive blood smear and high *P*.*falciparum* parasitaemia with lead levels classified in quartiles, whereas Tables 5 and 6 refer to the possibility of having elevated BLL.

**Table 3:** Logistic regression on the possibility of having a positive blood smear at 12 months.

Factor	AOR (95% CI)	p-value
Blood lead levels (BLL; μg/L)	(1st quartile = reference)	
BLL in the 2nd quartile	0.66 (0.22; 2.03)	0.47
BLL in the 3rd quartile	0.24 (0.06; 1.00)	0.05
BLL in the 4th quartile	0.19 (0.04; 0.95)	0.04
Ferritin levels (ferritin (mg/L) corrected on inflammation)	1.00 (1.00; 1.01)	0.03
Vitamin B12 (ng/mL)	0.99 (0.99; 1.00)	0.5
Low socio-economic status	1.81 (1.07; 3.07)	0.03
Prob>chi2 = 0.0002 Number of observations = 197		

**Table 4:** Negative binomial regression on factors associated with *P*.*falciparum* density (logarithm of parasite density at lead assessment).

Factor	Coefficient (95% CI)	p-value
Blood lead levels (BLL)	(1st quartile = reference)	
BLL in the 2nd quartile	-1.18 (-2.99; 0.64)	0.20
BLL in the 3rd quartile	-2.30 (-4.43; -0.18)	0.03
BLL in the 4th quartile	-2.10 (-4.00; -0.23)	0.03
Low socio-economic status	0.60 (-0.04; 1.24)	0.06
Number of observations = 197		

**Table 5:** Logistic regression on the possibility of having a positive blood smear at 12 months.

Factor	AOR (95% CI)	p-value
Elevated blood lead levels (BLL>5 μg/dL)	0.38 (0.15; 0.99)	0.048
Ferritin levels	2.86 (1.13; 7.27)	0.03
(logartihm of the ferritin (mg/L) corrected on inflammation)		
Low socio-economic index	1.42 (1.2; 7.93)	0.16
Inflammatory process (CRP levels ≥5 mg / mL)	3.09 (1.2; 7.93)	0.02
Prob>chi2 = 0.0005 Number of observations = 197		

**Table 6:** Linear regression on factors associated with *P*.*falciparum* density at 12 months (logarithm of parasite density at lead assessment).

Factor	Coefficient (95% CI)	p-value
Elevated blood lead levels (BLL>5 μg/dL)	-1.42 (-2.83; -0.02)	0.03
Low socio-economic index	0.43 (-0.16; 1.02)	0.15
Prob> = chibar2 = 0.00 Number of observations = 197		

In multivariate analysis, high lead levels were significantly associated with reduced risk of a positive blood smear and *P*. *falciparum* parasite density in logistic and negative binomial regression models, respectively. More precisely, infants with BLL in the 3^rd^ and 4^th^ quartile were significantly less likely to have a positive blood smear at 12 months (OR = 0.24, p-value = 0.05, and OR = 0.19, P-value = 0.04, respectively). Indeed, no positive blood smear was found among infants with lead poisoning. With regard to *Plasmodium falciparum* parasitemia, infants with BLL in the 3^rd^ and 4^th^ quartile were significantly less likely to have a high parasite density at 12 months (beta coefficient = -2.3, p-value = 0.03, and beta coefficient = -2.1, P-value = 0.03, respectively). Furthermore, infants with elevated BLL (i.e. BLL> 5 μg/ dL, Table 4) were significantly less likely to have a positive blood smear and a high *P*. *falciparum* density (AOR = 0.38 95% CI (0.15; 0.99), and beta coefficient = -1.42, P-value = 0.05, respectively). Factors associated with increased malaria risk include also high iron levels. In effect, elevated ferritin levels corrected on inflammation were associated with increased risk of a positive blood smear, also in the analysis of elevated lead levels (Table 5). In addition, low socio-economic status was also statistically associated to an increased malaria risk (AOR = 1.81, P-value = 0.03; Table 3).

Adjustments on known prognostics factors for malaria were included in the models to avoid potential confounders. However, they were not statistically significant and, consequently, they have been removed from the final model.

## Discussion

The high proportion of infants with elevated BLL (63%) and lead poisoning (19%) plead for the necessity of considering the possible influence of lead levels on the infant infectious morbidity, especially with regard to malaria, the main cause of mortality in children<5 years. In effect, high BLL were significantly associated with reduced malaria risk with regard to both the possibility of having a positive blood smear and *P*. *falciparum* density. Concern has been repeatedly raised up on the importance of alarmingly high anemia rates in West Africa[18], and both malaria and elevated BLL are associated with increased anemia rates.

Similar prevalence of elevated BLL has been found in other West-African regions. The mean BLL value for this study (7.4 μg/dL) is slightly lower than the mean BLL found by Nriagu et al in Nigeria in 2008[11] (8.9 μg/dL). In Jos, Nigeria, another study reported an average BLL of 11.2 μg/dL (range: 9.1–13.3 μg/dL), and that 55% had BLLs above 10 μg/dL[19]. Indeed, the existing epidemiological evidence reveals the high prevalence of elevated BLL among African infants. However, there is very limited evidence on their effect on malaria. Nriagu et al described the inverse association of BLL and malaria in univariate analysis (P-value = <0.001). Our results not only ascertain this association in univariate analysis, but they evidence the significant association of BLL with malarial risk taking into account complementary malaria risk factors. These results show that elevated BLL are also associated with reduced probability of a positive blood smear as well as reduced *P*.*falciparum* parasite density. As a consequence, epidemiological evidence in our study rejects the possible synergistic effect of lead on *P*.*falciparum* infection, but rather suggests a protective effect. In addition, the high BLL present in our sample raise concern on their possible harmful consequences for the infant health.

Indeed, sources of lead should be further investigated to implement public health policies targeting the reduction of the exposure to lead. In our case of study, sources of lead may include paint, piped water, leaded gazoline or consumption of animals killed by ammunition. These hypotheses are currently under study.

The mechanism by which lead might influence malaria infection has not been elucidated so far. However, Nriagu postulated that there are multiple levels at which lead can modulate the specific host response to *Plasmodium* infection including alterations in heme synthesis, immunoregulation, and iron metabolism.

Lead concentrates in red blood cells (RBC) in the context of lead poisoning[20]. The accumulation of lead in the RBC may inhibit the development of the parasite. Elevated intra-erythrocytic concentration of lead may interfere with the development from the ring form to the schizont stage and, consequently, lead exposure may be associated to reduced parasitemia in malaria-infected infants.

In addition, elevated BLL can exert a general effect on the immune regulatory function[21,22]. In this respect, both lead poisoning and malaria favor the cytokine response which, in turn, has an influence on the Th1/Th2 balance[23,24]. Indeed, a certain protection against severe malaria has been described as a consequence of the Th2 response following the alteration of the immune system induced by lead poisoning[25].

Alternatively, iron deficiency and hemoglobinopathies can foster the anti-parasite effect of lead in the context of the blood stages of *P*. *falciparum*. Indeed, iron deficiency may interfere with the proper use of iron by the parasite[26]. However, iron deficiency was not significantly correlated with malaria risk in our analyses. Finally, high intra-erythrocyte lead concentration can inhibit protein synthesis[27], and thereby interfere with the correct iron utilization by *Plasmodia*[26].

With regard to iron levels, high iron levels have already been associated with increased malaria morbidity[28]. This raises the concern on the iron supplements recommended by the WHO when anemia prevalence >40%, which is the case of Benin. Iron deficiency has frequently been linked to a certain protection against malaria[29]. Nevertheless, results on the effect of iron levels on malaria differ in the context of clinical trials with iron supplements. In a specific Cochrane review[29] no significant difference in clinical malaria episodes was detected between children supplemented with iron alone and those receiving a placebo (risk ratio (RR) = 0.99, 95% CI (0.90; 1.09). However, solid preventive measures against malaria were implemented in the clinical trials. Moreover, an increased risk of malaria with high iron levels was observed in trials that did not provide malaria surveillance and treatment, and the risk of malaria parasitaemia was higher with high iron levels (RR = 1.13, 95% CI (1.01; 1.26)[29]. Furthermore, published literature reports both iron and lead have a significant effect not only on malaria, but also on anemia. Indeed, strategies to tackle anemia should consider not only iron supplementation but, as said, public health policies should also imply the sources of elevated BLL.

Our study is subjected to certain limitations. In a cross-sectional design one can only find evidence of associations. Our hypothesis is that high lead levels may reduce malaria risk. But the sense of the association might be set into question. However, reverse causality seems unlikely. Malaria entails anemia, and this should facilitate the absorption of lead, while our results show the opposite. Another limit of cross-sectional designs is that they do not allow the study of temporality. As a consequence, this association should be further studied in the context of a prospective longitudinal follow-up with repeated measures. Indeed, it would be interesting to analyze the evolution of the association through infancy. In conclusion, exposure to lead can in no way be considered as a potential method to prevent malaria in children. Therefore, public health interventions should look forward to minimize infant exposure to lead by all possible means, along with a reinforcement of malaria preventive interventions among the pediatric population.

## Conclusion

In conclusion, our study shows that BLL are negatively associated with malarial risk considering other risk factors. Malaria entails high morbidity and mortality rates among infants under 5 years worldwide[10], and lead poisoning is the 6th most important contributor to the global burden of diseases measured in disability adjusted life years (DALYs) according to the Institute of Health Metrics, with Sub-Saharan African countries being predominantly responsible for the global DALYs[30]. Our study shows very high rates of EBLL in young infants (12 months). This should raise the awareness of public health authorities to further evaluate the sources and the epidemiological consequences for infants. Lead poisoning entails severe consequences for the development of the children and is associated with major health problems highly prevalent in West Africa, such as anemia and under-nutrition, having an important impact on the infants and their communities. In addition, our study is the first to show a negative association between lead levels and malaria risk in multivariable analyses. Therefore, these results should be confirmed in the context of a prospective cohort. Furthermore, the social and financial consequences of EBLL and lead poisoning appeal to further explore the epidemiological evidence of the association of EBLL with communicable diseases in developing countries. Finally, public health interventions should look forward to minimize the exposure to lead. Therefore, it is crucial to investigate the lead sources to better protect the population in West Africa.

## Supporting Information

S1 File Supporting information: TOVI database. (PDF)
