# Supplementary material for: Elevated Blood Lead Levels Are Associated with Reduced Risk of Malaria in Beninese Infants
Source: PLoS One. 2016 Feb 11;11(2):e0149049. doi: 10.1371/journal.pone.0149049 (PMC4751084; doi:10.1371/journal.pone.0149049)
Supplement: S1 File — (PDF) [file pone.0149049.s001.pdf]

datepb	pbfinal	pbfinal_cat
11/5/11	70	3
15/6/11	41	2
2/5/11	56	2
13/5/11	140	4
11/5/11	26	1
10/5/11	53	2
1/6/11	85	3
20/5/11	92	4
11/5/11	58	2
20/5/11	140	4
13/5/11	71	3
23/5/11	100	4
24/5/11	40	1
24/5/11	60	3
27/5/11	35	1
17/6/11	100	4
21/5/11	44	2
14/6/11	130	4
8/6/11	82	3
7/6/11	82	3
26/5/11	75	3
25/5/11	260	4
8/6/11	80	3
6/6/11	260	4
27/5/11	30	1
10/6/11	40	1
6/6/11	22	1
6/6/11	150	4
8/6/11	52	2
10/6/11	63	3
18/6/11	32	1
2/6/11	92	4
6/6/11	54	2
15/6/11	37	1
2/6/11	91	4
14/6/11	180	4
14/6/11	140	4
15/6/11	51	2
22/6/11	42	2
15/6/11	47	2
13/6/11	140	4
23/6/11	31	1
9/6/11	150	4
8/6/11	47	2
7/6/11	39	1
27/6/11	86	3
16/6/11	41	2
8/6/11	69	3
23/6/11	19	1
27/6/11	24	1
6/6/11	63	3

8/6/11	70	3
6/6/11	30	1
15/6/11	240	4
8/7/11	110	4
23/6/11	30	1
29/7/11	54	2
30/6/11	31	1
23/6/11	76	3
13/7/11	110	4
27/6/11	37	1
22/6/11	64	3
11/7/11	87	3
5/7/11	24	1
29/6/11	54	2
8/7/11	75	3
22/6/11	110	4
30/4/11	120	4
5/7/11	39	1
6/7/11	27,8	1
7/7/11	35	1
1/7/11	78	3
16/7/11	85	3
1/7/11	35	1
13/7/11	53	2
5/7/11	60	3
7/7/11	17	1
11/7/11	33	1
18/7/11	23	1
14/7/11	73	3
18/8/11	40	1
14/7/11	50	2
3/8/11	100	4
28/7/11	92	4
18/7/11	85	3
2/7/11	58	2
4/8/11	39	1
26/7/11	65	3
4/8/11	85	3
19/7/11	35	1
18/7/11	8,3	1
13/7/11	57	2
20/7/11	24	1
21/7/11	36	1
21/7/11	160	4
16/7/11	29	1
25/7/11	39	1
29/7/11	35	1
22/7/11	504	4
27/7/11	51	2
21/7/11	63	3
1/8/11	72	3
1/8/11	110	4

28/7/11	60	3
1/8/11	78	3
29/7/11	39	1
1/8/11	32	1
25/7/11	55	2
24/8/11	230	4
30/7/11	38	1
8/9/11	33	1
4/8/11	63	3
9/8/11	97	4
9/8/11	52	2
30/7/11	43	2
27/7/11	20	1
4/8/11	52	2
10/8/11	71	3
27/7/11	39	1
7/8/11	64	3
9/8/11	81	3
15/11/11	64,2	3
27/7/11	57	2
27/7/11	130	4
4/8/11	49	2
2/9/11	100	4
14/8/11	54	2
10/8/11	31	1
10/8/11	33	1
17/8/11	120	4
26/8/11	87	3
29/8/11	110	4
10/8/11	53	2
9/8/11	88	3
23/8/11	56	2
29/8/11	51	2
22/8/11	16	1
5/9/11	70	3
23/8/11	56	2
26/8/11	93	4
22/8/11	64	3
26/8/11	95	4
31/8/11	34	1
25/8/11	46	2
26/8/11	99	4
12/8/11	57	2
29/8/11	100	4
7/9/11	36	1
23/8/11	59	2
31/8/11	61	3
2/9/11	23	1
2/9/11	66	3
7/9/11	40	1
29/8/11	110	4
27/10/11	126	4

13/10/11	49,1	2
17/11/11	37,9	1
31/10/11	41	2
25/11/11	28,6	1
5/12/11	76,3	3
21/12/11	88,5	4
21/12/11	66,1	3
30/11/11	578	4
19/12/11	60,9	3
18/1/12	51,6	2
5/1/12	47,5	2
5/1/12	88,5	4
3/1/12	105	4
25/1/12	59,7	2
11/1/12	34	1
11/1/12	42,5	2
23/1/12	176	4
25/1/12	114	4
6/2/12	33,2	1
2/2/12	72,7	3
27/2/12	102	4
15/2/12	21,1	1
3/3/12	43,1	2
7/3/12	40	1
16/3/12	29	1
20/3/12	49,9	2
27/3/12	117	4
6/3/12	30,3	1
28/3/12	53,7	2
4/4/12	253	4
19/4/12	86,4	3
17/4/12	82,1	3
10/5/12	168	4
24/4/12	21,3	1
4/5/12	39,4	1
21/5/12	36,1	1
18/5/12	73,4	3
14/5/12	136	4
9/5/12	66,1	3
8/5/12	33,6	1
11/5/12	49,5	2
11/5/12	76,3	3
22/5/12	57,2	2
16/5/12	50,6	2
31/5/12	62	3
30/5/12	105	4
25/5/12	52,4	2
25/5/12	44,8	2
